# Probing the posture with machine learning provides physiological evidence supporting the enhanced body awareness hypothesis in trait mindfulness

**DOI:** 10.3389/fphys.2022.915134

**Published:** 2022-09-02

**Authors:** Charles Verdonk, Marion Trousselard, Takfarinas Medani, François Vialatte, Gérard Dreyfus

**Affiliations:** ^1^ Department of Neurosciences and Cognitive Sciences, Unit of Neurophysiology of Stress, French Armed Forces Biomedical Research Institute, Brétigny-sur-Orge, France; ^2^ ESPCI Paris – PSL University, Paris, France; ^3^ French Military Health Service Academy, Paris, France

**Keywords:** mindfulness, body awareness, posture, machine learning, proprioception

## Abstract

Enhanced body awareness has been suggested as one of the cognitive mechanisms that characterize mindfulness. Yet neuroscience literature still lacks strong empirical evidence to support this claim. Body awareness contributes to postural control during quiet standing; in particular, it may be argued that body awareness is more strongly engaged when standing quietly with eyes closed, because only body cues are available, than with eyes open. Under these theoretical assumptions, we recorded the postural signals of 156 healthy participants during quiet standing in Eyes closed (EC) and Eyes open (EO) conditions. In addition, each participant completed the Freiburg Mindfulness Inventory, and his/her mindfulness score was computed. Following a well-established machine learning methodology, we designed two numerical models per condition: one regression model intended to estimate the mindfulness score of each participant from his/her postural signals, and one classifier intended to assign each participant to one of the classes “Mindful” or “Non-mindful.” We show that the two models designed from EC data are much more successful in their regression and classification tasks than the two models designed from EO data. We argue that these findings provide the first physiological evidence that contributes to support the enhanced body awareness hypothesis in mindfulness.

## Introduction

Enhanced body awareness has been suggested as a cognitive mechanism through which mindfulness may improve health and well-being (Hölzel et al., 2011; Gu et al., 2015; Verdonk et al., 2020). Yet objective evidence, including physiological data, to support this hypothesis remains weak [for a review, see (Treves et al., 2019)]. Body awareness may be operationalized as the individual ability to feel engaged by information coming from the body and noticing subtle changes therein (Mehling et al., 2009). Proprioception, which refers to internal representation of the body in relation to space and movement, results from the cerebral integration of visual information and bodily signals that originate from within vestibular and somatosensory systems (Forbes et al., 2018; Tuthill and Azim, 2018). In the present study, the posture was investigated as a potential proxy for enhanced proprioception in trait mindfulness.

Previous work has reported mixed evidence for the association between mindfulness and measures of posture. Of note, here the term “mindfulness” includes dispositional trait (i.e., an ability to be mindful in daily life without any practice of mindfulness) and mindfulness intervention. Two studies reported that mindfulness, including mindfulness intervention and dispositional trait mindfulness, was associated with more stable postural balance (Mills and Allen, 2000; Rosenstreich et al., 2018). One study showed that a very brief mindfulness induction (6 min) does not significantly affect postural balance (Kee et al., 2012). Interestingly, mindfulness is also characterized by enhanced self-regulation of attention (Hölzel et al., 2011; Verdonk et al., 2020), which primarily involves attentional focus on bodily signals [specifically the breath (Lutz et al., 2015)]. In the literature, the contribution of attention to postural balance is supported by the association between poorer attention domain, as experimentally induced by the dual-task paradigm, and postural imbalance [for a review, see (Amboni et al., 2013)]. A few studies have shown that internal-focused attention induced by experimental instructions influences some measures of posture but not all (Vuillerme and Nafati, 2007; Rhea et al., 2019). It should be noted that the aforementioned studies mostly investigated a relatively small number of postural measures, thus suggesting that potential effects of mindfulness and attention on postural measures remain largely unknown.

Our work is grounded in the global neural workspace theory suggesting that conscious perception involves top-down attentional amplification, which amplifies sensory information and allows its integration into the current, conscious context where it becomes available to other neural processes [for a review of the global neural workspace theory, see (Dehaene and Changeux, 2011)]. Given that mindfulness is characterized by enhanced attentional skills (notably the self-regulation of attention) (Hölzel et al., 2011), we suggest that mindfulness could support the top-down process of attentional amplification, which in turn could facilitate the conscious processing of sensory information, including bodily signals (Verdonk et al., 2020). In other words, mindfulness could help overcome the situation in which information from the body remains unconscious by facilitating conscious access to this information, thus ultimately leading to enhanced body awareness. Regarding the posture, control processes mostly operate unconsciously but individuals may be aware of action of postural balance and can volitionally control it when desired (Amboni et al., 2013; Forbes et al., 2018). In the present study, we assume that body awareness could be particularly important in standing posture as the control of spontaneous, postural oscillations requires tracking low-intensity bodily changes (Fitzpatrick and McCloskey, 1994; Forbes et al., 2018) (see Supplementary Material, section *The biomechanical modeling framework of standing posture*, for a description of the biomechanical framework of standing posture). In addition, we assume that body awareness is more strongly engaged in postural control when individuals are keeping their eyes closed during standing. Indeed, in such a condition, sensitive pathways of postural control loop only involve bodily signals from the vestibular and somatosensory systems, and cannot benefit from visual information.

In practice, static posturography is a simple and affordable technique to objectively and quantitatively assess postural control (Visser et al., 2008; Błaszczyk, 2016) (see Supplementary Material, section *The biomechanical modeling framework of standing posture*, for an illustrated description of static posturography). The first challenge researchers face when investigating the posture using posturography is the large number of features (more than 70) that can be extracted from the postural signal (in the time and frequency domains). Interestingly, 16 of these postural features have been characterized as individual-specific: they show both low *within*-subject variability and high *between*-subjects variability, and they contribute to discriminate every individual postural pattern when analysed with the stepwise method (Yamamoto et al., 2015). Given the trait mindfulness conceived as a personality-like trait refers to individual differences in characteristic patterns (Brown and Ryan, 2003; Tang et al., 2016), we assumed in the present work that these 16 individual-specific postural features could be of particular interest to investigate the relationship between the posture and the trait mindfulness. The second challenge is that the relation between physiological and psychological phenomena is inherently nonlinear (Cacioppo and Tassinary, 1990). To address these two challenges, we searched for machine learning based models that could estimate the trait mindfulness (response variable) from a set of input variables consisting of the postural features and pairwise products thereof (see the Method, section *Model design*, subsection *Cross-term computation* for a detailed description).

A machine learning based model is a mathematical, parameterized relation between a set of input variables and one response variable. From a theoretical standpoint, a stochastic model is postulated in the form *y* = *f* (**θ**, **x**) + *ε*, where **θ** is the vector of parameters of the model, **x** is the vector of variables, and *ε* is a random variable, usually with zero mean, which is supposed to model all sources of noise or disturbances in the process that generates the observed quantity *y*. *f* (usually called regression function) is a parameterized function belonging to a family of functions selected by the designer; if prior knowledge suggests that the process that generates *y* from **x** is multilinear, then *f* is chosen to be a multilinear function. If no prior knowledge is available, the regression function *f* is chosen in the family of universal nonlinear approximators such as polynomials or neural networks. The family of the regression function having been selected, the purpose of regression is the following: given a set of observations of **x** and *y*, estimate, by an algorithmic process called “training”, the numerical values $\hat{\theta }$ of the parameters for which the distance between the observations and the estimations is minimum. After estimating these values, the estimation $\hat{y}$ of the quantity *y* is obtained by the deterministic (non-stochastic) model $\hat{y}\;=\;f\left(\hat{\theta },\;x\right)$ , also called predictive model or predictor. If the model is fed repeatedly with the same set of values of **x**, it always provides the same estimation $\hat{y}\;$ *.* If no satisfactory deterministic model can be found by training, one can conclude that the experimental data do not support the validity of the postulated stochastic models. The number of parameters defines roughly the complexity of the model. The higher the model complexity (i.e., the higher the number of parameters), the more accurate the relation between the observed input variables and the observed response variable. However, an overly complex model would be unable to account for data that are not used for estimating the parameters of model, i.e., to generalize to previously unseen data. This “generalization ability” depends on the ratio of the model complexity to the size of the dataset. If this ratio is too low, the model fails to account for the relation between the input variables and the response variable. Conversely, if the ratio is too high (i.e., model with too many parameters given the size of the dataset), the model overfits the training data, i.e., performs very accurately on the training data but generalizes poorly on novel data (Geman et al., 1992). In summary, the challenge is to find, given the experimental data, a predictive model that accounts for the relation between the observed input variables and the observed response variable as accurately as possible, and that generalizes satisfactorily to data unseen during training. Such a model can be found if and only if there exists a stochastic relationship between the input variables and the response variable.

The models described in the present article were designed using a well-established methodology of machine learning, which comprises three main steps: (i) among the candidate variables derived from the individual-specific postural features, selection of the input variables that are relevant to account for the trait mindfulness, (ii) selection of the model that has the optimal complexity given the available data, and (iii) estimation of the generalization ability of the selected model.

In the present work, we tested the enhanced body awareness hypothesis in trait mindfulness by first examining whether one can find, by machine learning, a deterministic relation between trait mindfulness and posture. To this end, we tested whether the self-reported trait mindfulness could be estimated successfully from the postural signal, given the data available from a sample of 156 subjects. The existence of such a model would demonstrate the existence of a deterministic relation between trait mindfulness and posture. Secondly, we explored how the relationship between trait mindfulness and posture is affected by the experimental conditions. The postural signal was recorded in two conditions: the participants were instructed either to stand quietly while keeping their eyes open (EO condition), or to stand quietly while keeping their eyes closed (EC condition). As mentioned above, we assume that body awareness is more strongly engaged in postural control in the EC condition than in the EO condition, because only bodily signals (from vestibular and somatosensory systems) are available in the former condition, while the latter condition also involves visual information. Therefore, exhibiting a model designed from data collected in EC condition that estimates self-reported trait mindfulness from postural signal more accurately than a model designed from data collected in EO condition (all other things being equal), would provide support for the enhanced body awareness hypothesis in mindfulness.

## Methods

### Participants

One hundred and eighty-one healthy volunteer participants were initially recruited from three different units of the French Army: 111 participants belonged to the Ecole des fusiliers marins et commandos of the French Navy (EFMC, Lorient, France), 46 participants belonged to submarine crews from the French Navy (SC, Brest, France), and 24 participants belonged to the French armed forces biomedical research institute (IRBA, Brétigny-sur-Orge, France). Unlike the first two units (EFMC and SC) that included active-duty military service members only, participants from the IRBA unit were mainly (70%) civilian personnel of the French Army Ministry. The only criterion for inclusion was age (between 18 and 60 years). All participants reported no medication and no history of somatic disorders. After completion of the experiments, data related to 25 participants were discarded: four of them due to equipment failures, and 21 of them due to issues concerning identification either of participant or of experimental condition. Data from the remaining 156 participants (mean age: 26 years old, SD: 8.7; 138 males – 88%) were analyzed.

### Procedure

Data were collected at the three unit sites, independently of any military operation or training. After reviewing the study description, participants provided written informed consent. Then, they completed the Freiburg Mindfulness Inventory (FMI) as self-report questionnaire of dispositional trait mindfulness (Walach et al., 2006; Trousselard et al., 2010). Finally, the postural signal was collected using the FEETEST 6 platform (TECHNO CONCEPT^®^, France) in two conditions: eyes open (EO condition) and eyes closed (EC condition). Due to technical specifications of the FEETEST 6 platform, each sequence lasted 52 s. The participants were instructed to stand quietly with their arms hanging at their sides and head in a normal forward-facing position, while focusing on a stationary target located at eye level approximately 2 m away (in the EO condition).

### Data management

**FIGURE 1:**
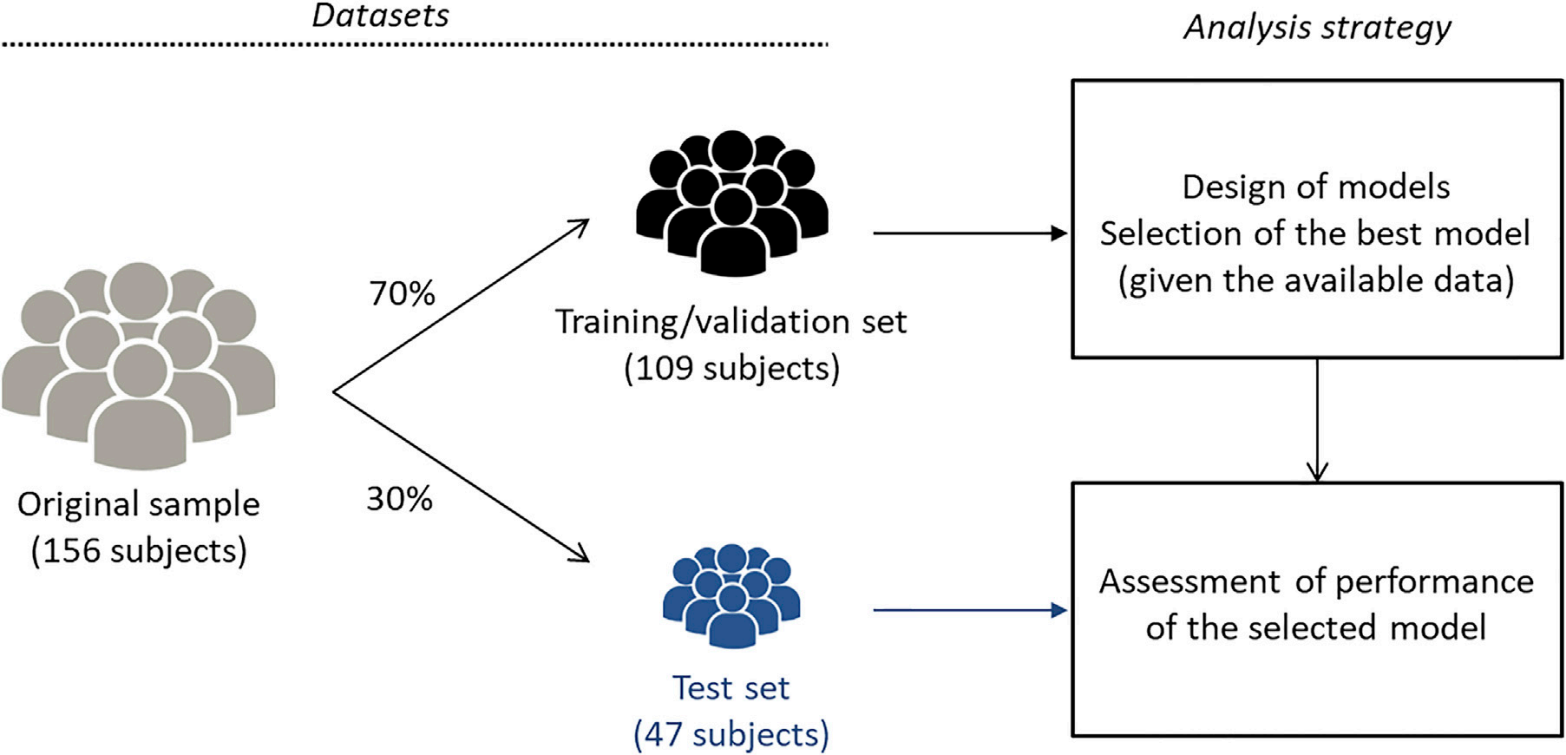
Flowchart of datasets and analysis strategy. The original sample included data from 156 participants. Models of different complexities were designed, and the best model (given the available data) was selected by leave-one-out cross-validation with the training/validation set, a subset of the original sample including 70% of the available examples (109 subjects). The performance of the selected models was assessed on the remaining 30% of the available examples (47 subjects). Thus, model training and selection were exclusively based on the training/validation set, while the assessment of the performance of the selected models was performed on a disjoint data set.

A subset of the data (the training/validation set) was used for designing and selecting the models; it included 70% of the available data (109 subjects). The performances of the selected models were assessed with the remaining 30% of the data (the test set, 47 subjects). This guarantees that performance assessment is carried out on data that are completely independent of the data used for training and selecting the models (Figure 1).

### Measures

#### Trait mindfulness

The 14-item FMI assesses dispositional trait mindfulness by indexing facets of Presence (i.e., being aware of all experiences in the present moment) and Non-judgemental acceptance (*i.*e., understanding that things are not necessarily how one wishes them to be). This questionnaire is semantically independent of a meditation context and it is applicable to all population groups, in particular to those with no practice of mindfulness training.

The questionnaire is scored using a four-point scale, with responses ranging from 1 (rarely) to 4 (almost always). A total mindfulness score was computed by adding the rating for all items, except for the 13th item that was reversely scored (Walach et al., 2006; Trousselard et al., 2010). The scale demonstrated acceptable levels of internal consistency in our sample (Cronbach’s α = 0.80). The total score of FMI was an integer quantitative variable, ranging from 14 to 56.

#### Postural sway

The FEETEST 6 (TECHNO CONCEPT^®^, France) includes four small independent platforms that measure the positions of the vertical ground reaction vector under the heel and the metatarsal, for each foot separately. Each of the four measurements is performed by three strain gauges with integrated rigid diaphragm positioned in a triangle facing heels and metatarsals. By averaging the positions of all the four vectors along antero-posterior and medio-lateral axes, the position of the Center of Pressure (CoP) of the whole body is computed. In other words, the CoP is the point of application of the vertical ground reaction force that is the sum of pressures acting on the part of the body in contact with the ground. During the recording, data were sampled at 40 Hz and information were transmitted to the data collection software (POSTUREWIN 4^©^) via a USB connection.

### Postural data processing

Postural data were first low-pass filtered with 10 Hz cut-off frequency using a fourth-order zero-phase-lag Butterworth filter. The values of the 16 individual-specific postural features were computed (Table 1; see Supplementary Material, section *Computation of postural features*, for the detailed computation of the postural features). In order to ensure that all features have the same order of magnitude, they were subsequently “z-score normalized” by subtracting their means and dividing by their standard deviation, so that each normalized feature had zero mean and unit standard deviation over the whole set of participants.

**TABLE 1:** The list of the 16 features that were extracted from the postural signal to characterize time-series of the center of pressure. A detailed description of the postural features, including their computation and their descriptive statistics, is provided in Supplementary Material (see Section *Computation of postural features* and Supplementary Table S1).

	Feature name	Description
1	MP3	Mean peak value on sway-density curve at R = 3
2	Mean-AP	Mean position of sway on AP axis
3	Mean-ML	Mean position of sway on ML axis
4	Zero-cross-V-AP	The number of zero crosses of low-pass filtered CoP velocity on AP axis
5	Beta-ML	Scale parameter of Gamma distribution fitted to the duration of mean CoP velocity crosses on ML axis
6	log-Alpha-ML	Log of shape parameter of Gamma distribution fitted to the duration of mean CoP velocity crosses on ML axis
7	log-Alpha-AP	Log of shape parameter of Gamma distribution fitted to the duration of mean CoP velocity crosses on AP axis
8	Beta-AP	Scale parameter of Gamma distribution fitted to the duration of mean CoP velocity crosses on AP axis
9	log-slope-MP	Log of slope of the line obtained by linear regression of mean peak values on sway-density curve vs. R from 2 to 5 mm
10	log-LNG	Log of total path length of CoP trajectory on the horizontal plane
11	log-MV	Log of mean CoP velocity
12	log-MV-ML	Log of mean CoP velocity on ML axis
13	log-MV-AP	Log of mean CoP velocity on AP axis
14	log-Power	Log of total power of CoP
15	log-Power-ML	Log of total power of CoP on ML axis
16	PF95AP	95% power frequency of CoP

R, radius of circle centered at the current CoP point (in mm); AP, anterior-posterior; ML, medio-lateral; CoP, center of pressure.

### Strategies for data analysis

#### Regression vs. classification approaches

The problem that we addressed was to find a deterministic relation, if any, between the postural signal and the trait mindfulness. To this end, we implemented two approaches for data analysis that need to be viewed as complementary: (1) a regression approach where the FMI score, as a quantitative variable, was predicted from the posture variables; and (2) a classification approach where the mindfulness status of subjects (“mindful” or “non-mindful”), as a categorical variable, was predicted from the posture variables*.* The mindfulness status was computed from the FMI score at the individual level using the group median as threshold: a subject was considered *mindful* if his/her total FMI score was larger than the median value reported in our original sample ( $\tilde{{\text{X}}_{FMI}}$ = 41), and *non-mindful* otherwise. Specifically, the median was preferred to the mean, because it guarantees that the number of subjects of the “non-mindful” class (i.e., subjects with mindfulness score smaller than the median) is equal to the number of subjects of the “mindful” class. This avoids the well-known, non-trivial problem raised by containing different numbers of subjects. Classical machine learning techniques were used to implement the regression and classification approaches. We show in the following that deterministic relations were found between the postural features and the FMI score (regression approach), and between the postural features and the mindfulness status as defined above (classification approach). A detailed description of regression and classification approaches is provided in the Supplementary Material, section *Strategy for data analysis*.

#### Linear and non-linear models

Multilinear regression and logistic regression were first performed because they are the simplest linear models for addressing regression and classification problems, respectively.

In addition to linear models, we used Neural Networks (NN) for addressing regression and classification problems (see Supplementary Material, section *Linear and nonlinear models*, for a detailed presentation of the machine learning techniques that were used, with emphasis on the NN model). NN models are a very popular family of universal nonlinear approximators that, when suitably designed, are particularly efficient for modeling nonlinear relationships, such as the relations between physiological and psychological phenomena (Cacioppo and Tassinary, 1990).

## Model design

As mentioned above, our purpose was to investigate whether it could be possible to find a machine-learning based model that could estimate, as accurately as possible, the trait mindfulness from the postural features, given the available experimental data. This problem was addressed in two steps: *(1)* among the candidate variables (postural features and pairwise products thereof), selection of the most relevant variables to estimate the trait mindfulness, and *(2)* selection of the model complexity that provides the best generalization ability, given the data used for training/validation. After variable and model selection, the performance of each selected model (regression model or classifier) was estimated on a separate dataset (the test set), disjoint from the training/validation set.

All steps described below were performed using Matlab 2018b (The Mathworks^®^), including the Statistics and Machine Learning toolbox, the Deep Learning toolbox and custom scripts.

### Variable selection

The purpose of variable selection is to identify irrelevant and/or redundant input variables that should be removed from the available data before designing models, in order to prevent overfitting (Guyon and Elisseeff, 2003).

The method used for selecting the appropriate set of variables for estimating the response variable of interest (either FMI score or mindfulness status) comprises three steps: (1) computing the candidate variables (postural features and pairwise products thereof), (2) ranking them in order of decreasing relevance to the response variable using the Orthogonal Forward Regression (OFR) algorithm, and (3) eliminating irrelevant candidate variables by the random probe method.

#### Cross-term computation

In addition to the 16 primary features, which were directly extracted from the postural signal, their pairwise products (except products of a primary feature by itself), called “cross-terms” (CT), were computed. Indeed, the product of two primary features may provide more relevant information than the two primary features separately. Thus, a total of 136 candidate variables were generated, including the 16 primary features and their 120 pairwise products.

#### OFR algorithm

**FIGURE 2:**
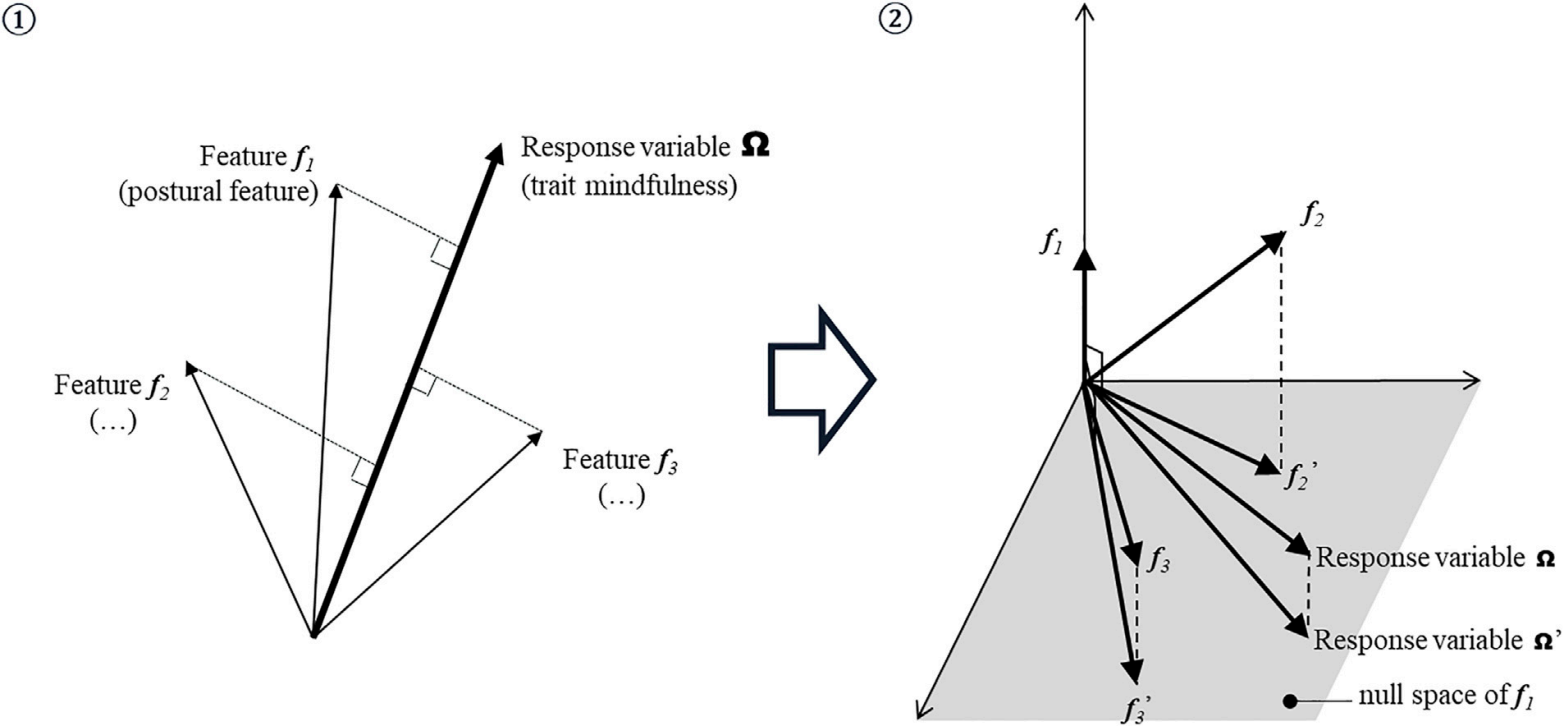
An illustration of the Orthogonal Forward Regression (OFR) algorithm for variable ranking. The algorithm operates iteratively: 1) all candidate variables are ranked according to their correlations to the response variable, namely the squared cosine of the angle between the candidate variable and the response variable; the most correlated variable (with the maximum squared cosine) is ranked first; 2) all remaining candidate vectors and the response variable are orthogonalized with respect to the first vector, in order to eliminate the contribution of the latter to the response variable; then, the first candidate variable is stored and removed from the set of candidate variables, and the algorithm iterates with the remaining orthogonalized candidate variables and the response variable, until all candidate variables are ranked.

The OFR algorithm (Chen et al., 1989) operates iteratively, given the candidate variable vectors ***f***_*k*_, *k* ϵ [1 … *N*] (*N* being the number of candidate variables) and the response variable vector ***Ω*** (Figure 2). All vectors are defined in observation space, whose dimension is equal to the number of examples.

1) All candidate vectors (including the “probes” defined in the next subsection) are ranked according to their correlations to the response variable. In observation space, the correlation *c*_*k*_ between the *k*-th candidate variable vector ***f***_*k*_ and the response variable vector is the squared cosine of the angle between these vectors: *c*_*k*_ = cos^2^ (***f***_*k*_, ***Ω***). The candidate variable that is most correlated to the response variable (with the maximum squared cosine) is ranked first.
2) All remaining candidate variables, and the response variable, are orthogonalized with respect to the first selected variable, in order to eliminate the contribution of the latter to the response variable. Then, the first selected variable is stored and removed from the set, and the algorithm is iterated with the remaining orthogonalized variables, until all candidate variables are ranked.

#### Random probe method

Variable selection was performed by the random probe method (Stoppiglia et al., 2003). Specifically, 100 randomly drawn “probe” vectors were appended to the set of candidate vectors, and ranked together with the latter as described above. The higher the probability for a probe to rank better than a non-probe candidate variable, the lower the relevance of the latter. For each non-probe candidate variable *i*, the cumulative probability $P_{p}\left(i\right)$ for a probe *p* to rank better than that variable was estimated; $P_{p}\left(i\right)$ can be interpreted as the risk of keeping candidate variable *i* although it is irrelevant. All non-probe candidate vectors for which that risk is larger than a user-defined threshold were discarded. The threshold was chosen to be 0.10 in the EC condition for regression and classification; in the EO condition, the thresholds were chosen to be 0.05 and 0.20 for regression and classification, respectively (see Supplementary Material for detailed explanations of the interpretation and the choice of the threshold).

### Model selection

The purpose of model selection is to select the model that is expected to generalize best, i.e., to have the smallest generalization error, given the available data. It was performed by Leave-One-Out (LOO) cross-validation, a method that provides an unbiased estimation of the generalization error of a model (Vapnik, 2013). Two steps were performed: (1) among models with the same complexity (e.g., NN models with the same number of hidden neurons), trained with different initial parameter values, find the NN model with initial parameter values that achieves the lowest generalization error; (2) among models with different levels of complexity (e.g., for classification, the logistic model and NN classifiers with a variable number of hidden neurons), find the model that achieves the lowest generalization error.

#### Jacobian matrix

As a preliminary step, we discarded models that were obviously prone to overfitting by checking the rank of their Jacobian matrices. The Jacobian matrix of a NN model with q  parameters $\left\{w_{1},\;\;w_{1},\;\ldots ,\;w_{q}\right\}$ , trained from a dataset of *N* examples, is the (*N*, *q*) matrix whose column *i* contains the values of the derivative of the model output with respect to parameter $w_{i}$ . Hence, each column of the Jacobian matrix expresses the effect of the variation of a parameter on the model output. The rank of the Jacobian matrix is the number of linearly independent columns, *i.e.* the number of parameters whose effects on the output are linearly independent. If the Jacobian matrix does not have full rank (*i.e.* if its rank is not equal to q ), it means that the effects, on the model output, of two parameters (or more) are not independent. In other words, the model has too many parameters because some of them are redundant, so that overfitting is very likely to occur. Such a model should be discarded (Zhou and Si, 1998; Dreyfus, 2005).

#### Root mean square error

One could intuitively consider that model selection should be based on the minimum of the Root Mean Square Error (RMSE) on the training/validation set (Equation 1), because it reflects the distance between the model predictions and the measured values of the response variable:

$$\text{RMSE}=\sqrt{\frac{1}{N}\sum_{k=1}^{N}{\left(r_{k}\right)}^{2}}$$ (1)

where $r_{k}$ is the modeling error (difference between the experimental value of the response variable and its estimated value) on example *k*, and where the summation is performed over all N examples of the training/validation set. However, the RMSE on the training/validation set is not a relevant estimator of the generalization error, because the modeling error on the training/validation set can be made as small as desired by just increasing the complexity of the model, which is detrimental to generalization. Thus, the value of the RMSE on the training/validation set is not a suitable criterion for model selection (Dreyfus, 2005).

#### Leave-one-out

Model selection was based on the comparison of the Leave-One-Out (LOO) score between candidate models of increasing complexity. For a given complexity, a LOO score is computed in *N* steps where *N* is the number of examples of the training/validation set (*n* = 109); at each step, one example of the training/validation set is withdrawn from the set, a model is trained (with a given set of initial parameter values) with the remaining *N*-1 examples; the modeling error on the withdrawn example is computed, and the LOO score of the model is computed as described below. In the present work, this procedure was iterated 300 times, with 300 different sets of initial parameter values. Finally, the model with the smallest LOO was stored as the “best” candidate model of the considered complexity, given the training/validation data. This procedure was performed for complexities (number of hidden neurons) ranging from 0 hidden neuron (i.e., multilinear model) to five hidden neurons, as described in the *Results* section.

For regression, the LOO score of a model is computed as (Equation 2):

$$S_{LOO}=\sqrt{\frac{1}{N}\sum_{k=1}^{N}{\left(r_{k}^{-k}\right)}^{2}}$$ (2)

where $r_{k}^{-k}$ is the modeling error on example *k* when it is withdrawn from the training/validation set, the model having been trained on the remaining *N*-1 examples (Dreyfus, 2005). Thus the LOO score is an estimation of the generalization error of the model, based on the training/validation data.

For classification, the LOO score is computed as follows: if the left-out example *k* is misclassified, the error *r*_*k*_^*-k*^ is taken equal to 1, otherwise to 0. The LOO score is computed as (Equation 3):

$$S_{LOO}=\frac{1}{N}\sum_{k=1}^{N}r_{k}^{-k}$$ (3)

The sum on the right-hand side is the number of misclassification errors, hence the LOO score is an estimation of the misclassification rate of the classifier, given the training/validation data.

### Assessment of the model performance

#### Regression approach

Two indices were computed for model assessment: (i) the RMSE on the test set, disjoint from the training/validation set, and (ii) the Pearson correlation coefficient between the model estimations and the measured values of the FMI score. The RMSE on the test set (“test RMSE”) is computed as in Equation 1, but for the fact that the data are those of the test set. The lower the test RMSE, the better the model performance on data unseen during the design of the model. The Pearson correlation coefficient provides an additional information, namely, to what extent the estimations and the measured values are linearly related; however, this quantity alone is not a valid assessment of model performance, as a very poor model (with a very high test RMSE) may have a correlation coefficient of 1 (e.g., a model that would always provide an estimation equal to 1000 times the measured value). In addition to the aforementioned indices, it is useful to compare the test RMSE of the selected model with the RMSE of a “baseline model” in order to ascertain that the results are acceptable. In the regression approach, the baseline model is the simple-minded model that estimates the FMI scores of all examples of the test set as equal to the mean FMI score on the training/validation set.

#### Classification approach

When addressing a classification problem with two classes (mindful and non-mindful), model performance can be assessed in a number of ways.

#### ROC curve and related indices

Performance indices classically include (i) the accuracy (i.e., percentage of examples that are correctly classified, Equation 4) or, equivalently, the misclassification rate (1-accuracy), (ii) the sensitivity (i.e., the percentage of examples of the *mindful* class – considered as the “positive” class – that are correctly classified, also called true positive rate, Equation 5), and (iii) the specificity (i.e., the percentage of examples of the *non-mindful* class that are correctly classified, Equation 6):

$$\text{Accuracy}=\frac{\text{True}\;\mathrm{mindful}\;\text{examples}\;+\;\text{True}\;non-mindful\;\text{examples}}{\text{Total number of examples}}$$ (4)

$$\text{Sensitivity}=\frac{\text{True}\;mindful\;\text{examples}}{\text{Total number of}\;mindful\;\text{examples}}$$ (5)

$$\text{Specificity}=\frac{\text{True}\;non-mindful\;\text{examples}}{\text{Total number of}\;non-mindful\;\text{examples}}$$ (6)

The Receiver Operating Characteristic (ROC) curve depicts graphically the relation between sensitivity and (1−specificity) (i.e., percentage of examples of the “non-mindful” class that are (incorrectly) assigned to the “mindful” class, also called false positive rate), at various thresholds. Each threshold value represents the decision boundary, within the range [0,1] , for predicting whether an example belongs to the “mindful” class: for instance, an example is classified as “mindful” if the corresponding output of the model is above threshold, otherwise it is considered “non-mindful”. The area under the ROC curve (AUC) quantifies the ability of the model to correctly assign an example to the “mindful” class. A value of 1 denotes perfect classification performance, whereas a value below 0.5 means that the model does not perform better than a random classifier (defined below) (Bradley, 1997).

#### Random classifier

In addition to computing the above performance indices, it is useful to compare the results of the selected classifier with those of a simple-minded “baseline classifier”. In the present work, we investigated whether one can find, by machine learning, a deterministic relation between the posture and the trait mindfulness; specifically, in the classification approach, we tested whether mindfulness status (mindful vs*.* non-mindful) can be inferred from the selected variables. If posture and trait mindfulness were unrelated, the selected model should not perform better than chance. In the present work, the two classes have the same number of examples; thus, the most suitable baseline classifier is a random classifier that assigns each example to one of the two classes with equal probability, hence has 50% accuracy. Any classifier whose accuracy is smaller than 50% is not acceptable.

## Results

### Overall summaries

Table 2 summarizes the basic statistics on demographic (age and gender) and biometric (weight and height) measures for the three military units where examples originated from, and for the two subsets of data we created from the original sample (training/validation and test sets).

**TABLE 2:** Summary of demographic (age and gender) and biometric (weight and height) data for the three military units from which the examples originated. These data pertain to the 156 subjects selected from the study (see *Participants* section).

	Variables							
	Age (years)		Gender (males)		Weight (kg)		Height (cm)	
	M	SD	n	%	M	SD	M	SD
Total (*n* = 156)	26	9	138	88	74	10	177	8
Military unit								
EFMC (*n* = 99)	22	3	99	100	74	7	178	6
SC (*n* = 33)	28	7	31	94	78	13	177	8
IRBA (*n* = 24)	40	12	8	33	67	12	169	8

N, number of subjects; M, mean; SD, standard deviation; n, number of males; %, proportion of males; EFMC, ecole des fusiliers marins et commandos; SC, submarine crew; IRBA, french armed forces biomedical research institute.

Table 3 reports the score of trait mindfulness, as assessed with the FMI questionnaire, for the three military units. For the variable “mindfulness status” (see *Method* section for a description of how it was computed), 50% of examples were labelled as “mindful” and that proportion was similar in the training/validation (*N*_*mindful*_ = 53) and test sets (*n*_*mindful*_ = 23).

**TABLE 3:** Score of trait mindfulness, as assessed with the Freiburg Mindfulness Inventory, for the three military units from which the examples originated. These data pertain to the 156 subjects selected from the study (see *Participants* section).

	Freiburg mindfulness inventory	
	M	SD
Total (*n* = 156)	41	6
Military unit		
EFMC (*n* = 99)	43	5
SC (*n* = 33)	42	5
IRBA (*n* = 24)	37	7

N, number of subjects; M, mean; SD, standard deviation; EFMC, ecole des fusiliers marins et commandos; SC, submarine crew; IRBA, french armed forces biomedical research institute.

### Selected variables

The variables that were selected as relevant to estimate the trait mindfulness (as described in the *Variable Selection* section) are presented in Table 4. Only cross-terms (pairwise products of primary features), were selected with the chosen thresholds. Variables that were selected varied depending on the condition (EC or EO), but they were the same for classification and regression.

**TABLE 4:** Results of variable selection for Eyes closed and Eyes open conditions. The selected variables were the same for regression and classification. A detailed description of the postural features, including their descriptive statistics, is provided in Supplementary Material (see Section *Computation of postural features* and Supplementary Table S1).

	Selected variables
Eyes Closed condition	Mean-ML × log-Alpha-AP
	Mean-ML × PF95-AP
Eyes Open condition	MP3 × Beta-AP

### Selected models

#### Regression approach

**FIGURE 3:**
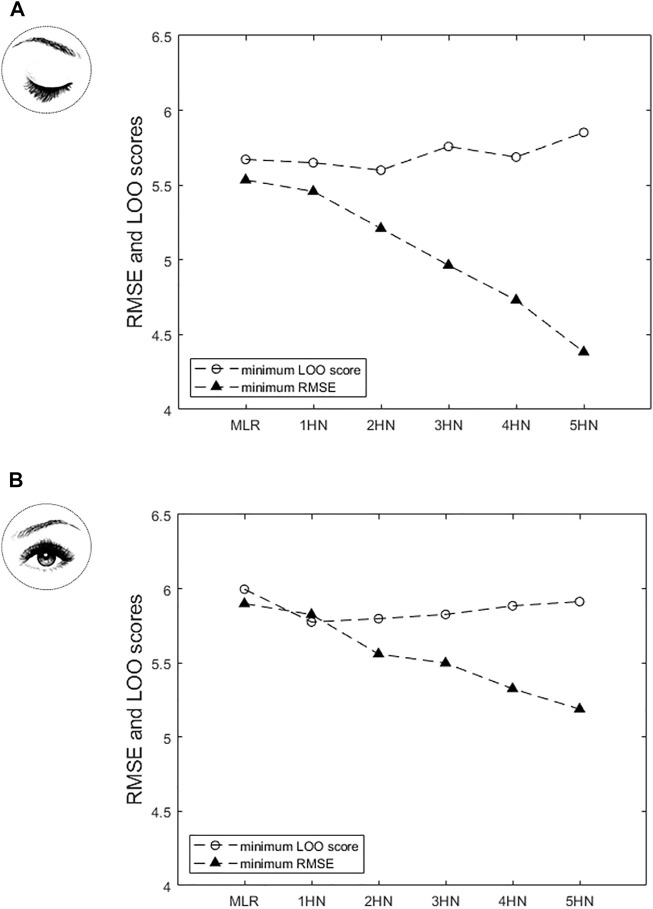
In the regression approach, variation of the Leave-One-Out (LOO) score and of the Root Mean Square Error (RMSE) on the training set as a function of model complexity [MLR: multilinear regression model; *n*HN: neural network (NN) with *n* hidden neurons (*n ≠* 0)], **(A)** in Eyes closed (EC) condition and **(B)** in Eyes open (EO) condition. In the EC condition, the LOO score is minimum with the NN model including two hidden neurons. Therefore, the NN model including two hidden neurons was selected to address the regression problem in the EC condition. In the EO condition, the LOO score is minimum with the NN model including a single hidden neuron. Therefore, the NN model including a single hidden neuron was selected to address the regression problem in the EO condition.

Figure 3 shows the LOO score and the training RMSE of the models on which the LOO method was applied (*i.e.* models whose Jacobian matrix had full rank) for the regression approach, in EC and EO conditions separately. As expected, the RMSE decreases when the model complexity (number of hidden neurons) increases, from the multilinear model (0 hidden neuron) upward. By contrast, the LOO score goes through a minimum for the NN model including two hidden neurons in the EC condition, and subsequently increases with increasing model complexity (Figure 3A). Therefore, the NN model including two hidden neurons was selected as the best model, given the experimental data, to address the regression problem in the EC condition. In the EO condition, the LOO score is minimum for the NN model including a single hidden neuron and remains higher with the multilinear model and other NN models (Figure 3B). Therefore, the NN model including a single hidden neuron was selected as the best model, given the experimental data, to address the regression problem in the EO condition.

#### Classification approach

**FIGURE 4:**
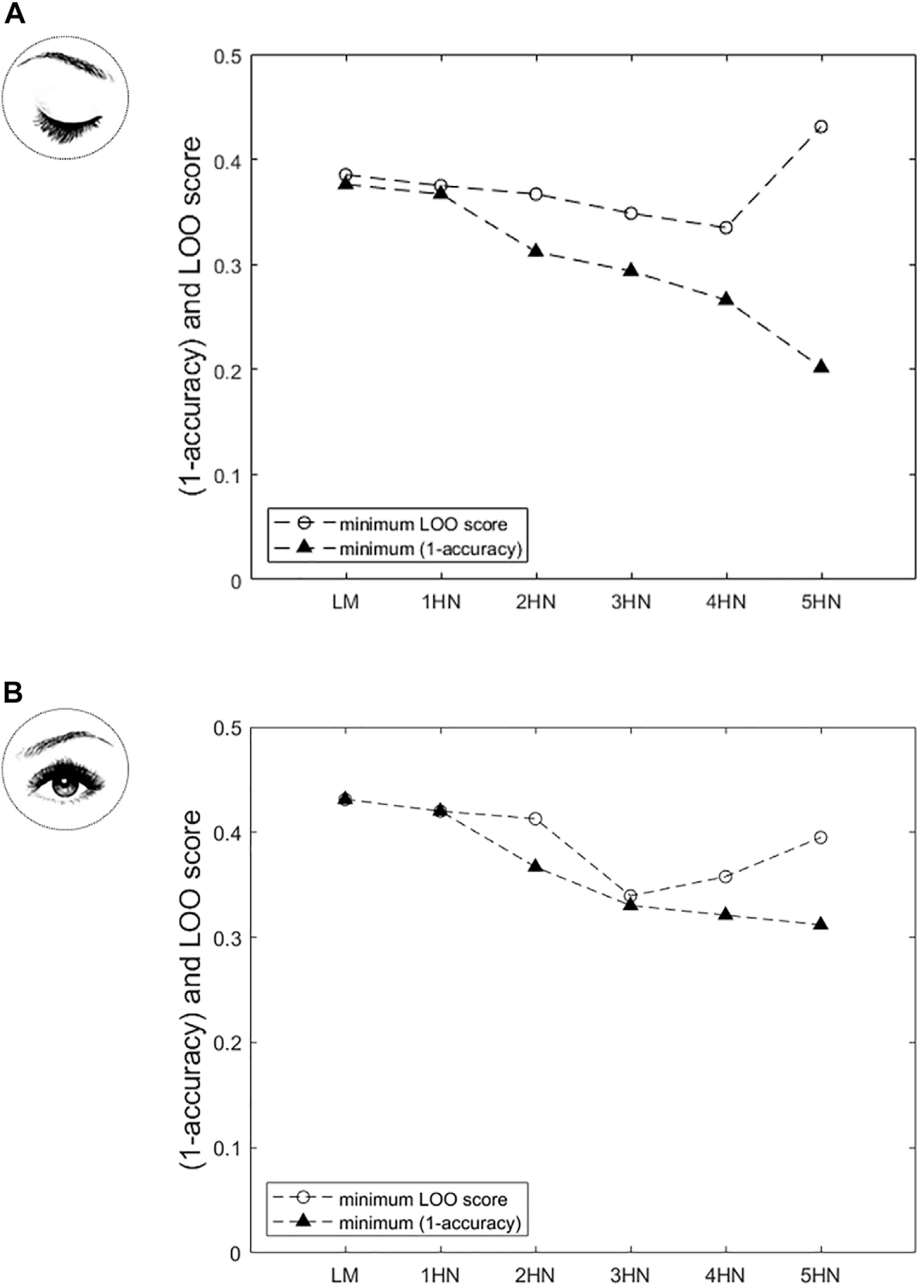
In the classification approach, variation of the Leave-One-Out (LOO) score and of the misclassification rate (1−accuracy) as a function of model complexity [LM: logistic regression model; *n*HN: neural network (NN) with *n* hidden neurons (*n ≠* 0)], **(A)** in Eyes closed (EC) condition and **(B)** in Eyes open (EO) condition. In the EC condition, the LOO score is minimum for the NN model including four hidden neurons. Therefore, the NN model including four hidden neurons was selected to address the classification problem in the EC condition. In the EO condition, the LOO score is minimum for the NN model including three hidden neurons. Therefore, the NN model including three hidden neurons was selected to address the classification problem in the EO condition.

Figure 4 shows the LOO score and the misclassification rate (1−accuracy) of the models on which the LOO method was applied (i.e., models whose Jacobian matrix had full rank) for the classification approach, in EC and EO conditions separately. As expected, the misclassification rate decreases when the model complexity increases, from the logistic model (0 hidden neuron) upward. By contrast, the LOO score goes through a minimum for the NN model including four hidden neurons, and increases for the NN model including five hidden neurons (Figure 4A). Therefore, the NN model including four hidden neurons was selected as the best model, given the experimental data, to address the classification problem in the EC condition. In the EO condition, the LOO score is minimum for the NN model including three hidden neurons and remains higher with the logistic model and other NN models (Figure 4B). Therefore, the NN model including three hidden neurons was selected as the best model, given the experimental data, to address the classification problem in the EO condition.

### Model performance

#### Regression approach

Table 5 shows the performance of the models that were selected for addressing the regression problem, in EC and EO conditions separately. In the EC condition, the training/validation RMSE and the test RMSE of the selected model are smaller than the test RMSE of the baseline model; in addition, the training/validation and the test RMSE are of the same order of magnitude, which shows that the test set is a representative sample of the available data. The estimated and measured values of the FMI score are significantly positively correlated, both on the training/validation set and on the test set. Taken together, these findings show that such a model is acceptable. Hence, we show that, given the data obtained in EC condition, there exists at least one deterministic function that provides a reliable estimation of the FMI score from the postural signal.

**TABLE 5:** Performance of the models that were selected for addressing the regression problem, in the Eyes closed condition and in the Eyes open condition. Two indices of performance are reported: *(i)* the Root mean square error (RMSE), and *(ii)* the Pearson correlation coefficient *ρ* between the model predictions and the measured values for the Freiburg Mindfulness Inventory score. Results are presented separately for the training/validation set and the test set, together with the results of the baseline model.

	RMSE	Pearson correlation *ρ* (p-value)
Eyes Closed condition (NN model including two hidden neurons)		
Training/validation set	5.21	0.46 (<0.001)
Test set	5.70	0.33 (<0.05)
Baseline model (test set)	5.98	0
Eyes Open condition (NN model including a single hidden neuron)		
Training/validation set	5.83	0.30 (<0.001)
Test set	5.87	-0.07 (0.66)
Baseline model (test set)	5.40	0

RMSE, root mean square error.

By contrast, in the EO condition, the test RMSE of the selected model is much larger than the test RMSE of the baseline model. Additionally, on the test set, the model predictions and the measured values for FMI score are uncorrelated. Taken together, these results show that the model is unacceptable. Hence, given the data obtained in EO condition, we have been unable to find a deterministic function that provides a reliable estimation of the FMI score from the postural signal.

#### Classification approach

The upper part of Table 6 shows the performance of the NN model including four hidden neurons for addressing the classification problem in the EC condition. The accuracy of the NN model on the test set (64%) is much higher than the performance of the baseline classifier that was defined with 50% accuracy (see the Method for detailed explanations). Hence, we show that, given the data obtained in EC condition, there exists at least one deterministic function that provides a reliable estimation of the mindfulness status from the postural signal.

**TABLE 6:** Performance of the NN model when addressing the classification problem in the Eyes Closed condition (upper part) and the Eyes Open condition (lower part). Results are reported separately for the training/validation set and the test set, together with the performance of the baseline model (random classifier).

	Performance indices	
	Accuracy (%)	AUC
Eyes Closed condition (NN model including four hidden neurons)		
Training/validation set	75	0.82
Test set	64	0.70
Eyes Open condition (NN model including three hidden neurons)		
Training/validation set	67	0.68
Test set	53	0.56
Baseline classifier	50	0.5

AUC, area under the ROC curve; NN, neural network.

The lower part of Table 6 shows the performance of the NN model including three hidden neurons for addressing the classification problem in the EO condition. The NN model has 67% accuracy on the training/validation set but the accuracy drops to 53% on the test set, which is very close to the performance of the baseline classifier (50%). Hence, given the data obtained in EO condition, we have been unable to find a deterministic function that provides a reliable estimation of the mindfulness status from the postural signal.

To summarize, regression and classification approaches showed that, given the available experimental data, models trained from postural data collected in EC condition, with the selected variables, perform correctly, while the models trained from postural data collected in EO condition do not, all other things being equal. Hence, in EC condition, at least one neural network model can be exhibited, which provides an accurate estimation of the FMI score from the postural signal, while this is not possible in EO condition.

## Discussion

In the present study, we aimed to investigate the posture as a proxy for enhanced body awareness (proprioception) in trait mindfulness. To this end, we examined whether the self-reported trait mindfulness could be estimated successfully from the postural signal, using machine learning based models and given the data available from a sample of 156 healthy subjects. Specifically, we tested whether the FMI score could be estimated from the postural signal (regression approach), and whether discrimination of mindful from non-mindful participants could be performed from the same signal (classification approach). The first observation is that NN models designed from postural data collected in EC condition successfully estimate the mindfulness status (with 64% accuracy), and the FMI score, from the selected postural features. Assuming that the selected models should perform better than their respective baseline model if postural signal accounts for self-reported trait mindfulness (see section *Assessment of the model performance* in the Method for detailed description of baseline model), our results support the existence of a stochastic relation between posture and trait mindfulness. We further explored how the level of body awareness engagement in postural control may affect this relationship, assuming that body awareness is more strongly engaged in postural control when the latter rests on body cues only (from vestibular and somatosensory systems) than when visual information is available. In our work, body awareness engagement in postural control was experimentally controlled by instructing participants either to keep their eyes closed (EC condition with *high* body awareness engagement) or to keep their eyes open (EO condition with *low* body awareness engagement) during standing. The second observation is that we found an effect of the experimental condition on model performance: a model trained from data collected in EC condition estimates the trait mindfulness from the selected postural features more accurately than a model trained from data collected in EO condition (classification: 64% vs. 53% accuracy; regression: presence vs. lack of correlation between predicted and measured FMI scores). This finding supports the idea that the strength of relationship between posture and trait mindfulness increases as a function of body awareness engagement in postural control. In summary, our results show that self-reported trait mindfulness and postural signal are associated, and that the higher the body awareness engagement in postural control the stronger the relationship between trait mindfulness and posture. Taken together, our findings provide the first physiological argument based on the postural signal that supports the hypothesis that trait mindfulness is characterized by enhanced body awareness (Hölzel et al., 2011; Treves et al., 2019; Verdonk et al., 2020).

Variable selection was performed to identify the set of relevant postural features for estimating the trait mindfulness. The most relevant variables were found to be cross-terms, i.e. pairwise products of the primary features that were extracted from the postural signal. This means that each primary feature of the product, taken separately, is not as relevant, for estimating the trait mindfulness, as a nonlinear combination of the two features such as – but not limited to – their product. It could be argued that the product of postural features, although relevant for estimating trait mindfulness by our machine learning models, remain difficult to interpret functionally and in terms of postural control. Future studies are needed to increase the biomechanical interpretability of the machine learning models developed in our study, which is a necessary requirement to make the enhanced body awareness (proprioception) hypothesis in mindfulness more readable in practice (e.g., for potential clinical use in mindfulness based programs). As a preliminary step, one could focus on the postural feature Mean-ML that was included in both selected combinations of primary features in the EC condition. Basically, the feature Mean-ML reflects the average position of the CoP along the medio-lateral axis over time. Considering that enhanced proprioception in mindful individuals should be associated with less displacements of their body’s center of pressure (i.e., better postural stability), one could hypothesise that mindful individuals have their CoP closer to the equilibrium point (the central point between both internal malleolus, which also corresponds to the origin of axes on statokinesigram; see Supplementary Material, section The biomechanical modeling framework of standing posture), compared to non-mindful individuals. In other words, one could hypothesise that mean and standard deviation values of the postural feature Mean-ML are closer to zero for mindful individuals than for non-mindful individuals. Interestingly, our data provide evidence, which need to be replicated with a larger sample, in favour of this hypothesis (see Supplementary Table S1 that includes statistics of the feature Mean-ML for mindful and non-mindful individuals separately).

Even though our findings provide preliminary physiological evidence supporting the enhanced body awareness hypothesis in mindfulness, they do not allow inferring any causal relationship between self-reported trait mindfulness and enhanced body awareness (proprioception). This limitation in the interpretation of results is important to consider if operationalization of mindfulness is extended to training in the form of brief interventions (e.g., Mindfulness Based Stress Reduction, Mindfulness-Based Cognitive Therapy) or long-term practice. Only studies that will experimentally manipulate mindfulness will provide evidence, if any, that mindfulness training may causally improve proprioceptive body awareness, as assessed with the analysis of posture. It should be noted that a causal, beneficial effect of mindfulness training on body awareness has been reported before using behavioural tasks [e.g., heartbeat tracking, tactile detection; see (Treves et al., 2019)], but needs to be extended to physiological signals originating from within sensory systems of the body.

Given the inherently complex (nonlinear) nature of psychophysiological relationships (Cacioppo and Tassinary, 1990), machine learning techniques were used for finding numerical models that can account for the relation between self-reported trait mindfulness and postural signal as accurately as possible, and that have the best generalization ability (i.e., that would perform similarly on novel data). We implemented a robust, proven machine learning methodology that was best suitable given our relatively small sample size, allowing us to reach our primary goal: generate physiological evidence that supports the enhanced body awareness hypothesis in mindfulness. Regarding potential clinical use of the present findings, our proof-of-concept study suggests that postural signal could be an interesting candidate physiological marker (i.e., a biomarker) of trait mindfulness. Yet, the development of a reliable predictive tool to objectively measure trait mindfulness by analysing the postural signal would primarily require a larger sample. If confirmed with a larger sample, our insights could have implications for future mindfulness research since trait mindfulness is usually assessed by means of self-report psychological scales (Sauer et al., 2013) that raise methodological concerns due to their vulnerability to limitations of introspection and social-desirability biases (Baumeister et al., 2007; Grossman, 2011; Van Dam et al., 2018).

From a psychophysiological perspective, the limited performance of our models might partially be due to fluctuations of body awareness engagement in postural control during recording. Indeed, unlike the psychological trait of mindfulness that is supposed to be relatively stable over time (Sauer et al., 2013), the subject’s postural control could have been contaminated by a range of psycho-cognitive processes (e.g., changes in attention, emotional thoughts, etc.) that are known to be associated with changes in postural signal (Amboni et al., 2013; Adkin and Carpenter, 2018). We encourage the investigation of the relationship that posture has with *state* (context specific) and *trait* (individual specific) components of mindfulness, for example by recording postural signal before and while participants are engaged in a mindfulness meditation training in a standing position. Furthermore, self-report measure of trait mindfulness relies on a verbal method that provides access only to introspective information that is consciously processed by the subject (Dehaene et al., 2006). On the other hand, a dominant portion of the postural control drive is subcortical, and conscious perception of postural control mechanisms remains limited (Forbes et al., 2018). We suggest that discrepancy between the *conscious* and (predominantly) *unconscious* nature of processes underlying the self-report of trait mindfulness and the postural control respectively might explain part of the limited performance of our psychophysiological model. Recently, we have proposed that cognitive functioning associated with mindfulness could be characterized with lower consciousness threshold that facilitates the conscious processing of information coming from within (body awareness and self-awareness) and outside the body (world awareness) (Verdonk et al., 2020). Interestingly, we observe that our model (in the classification approach) accounts for relation between trait mindfulness and posture more accurately in the mindful sample than in the non-mindful sample, as reflected by its greater sensitivity (70%) compared to its specificity (58%). This finding, which is preliminary and needs to be confirmed by a dedicated study, suggests that mindful functioning could potentially contribute to increase the proportion of postural control drive that is consciously processed.

Finally, in our study, analysis of postural signal focused on 16 primary features (and their cross-terms) that have been characterized as contributing to discriminate between individual postural patterns (Yamamoto et al., 2015). Yet, it should be noted that a much greater number of features (more than 70) can be extracted from the postural signal in the time and frequency domains. Future studies are encouraged to explore the high dimensionality inherent in postural data in using the existing wide range of machine learning techniques and algorithms, which could ultimately lead to better modelling of complex psychophysiological processes that characterize mindfulness.

## Code availability

The Matlab custom scripts used in the design of models reported in this paper are available from the corresponding author upon reasonable request.

## Data Availability

All requests for raw and analyzed data should be sent to dcssa-paris@sante.defense.gouv.fr, because they will be reviewed by our legal department (French Military Health Service) to verify whether the request is subject to any confidentiality constraints. Requests regarding materials, including programming code, should be sent to the corresponding author (CV).
