# Supplementary material for: Probing the posture with machine learning provides physiological evidence supporting the enhanced body awareness hypothesis in trait mindfulness
Source: Front Physiol. 2022 Sep 2;13:915134. doi: 10.3389/fphys.2022.915134 (PMC9480617; doi:10.3389/fphys.2022.915134)
Supplement: Supplementary file 1 [file DataSheet1.PDF]

Supplementary information

1) The biomechanical modeling framework of standing posture

Generally speaking, the ability of humans to stand requires a balance control, because standing erect is inherently an unstable situation. Two thirds of the body mass is located in the upper two thirds of the body, and, in addition to possible exogenous disturbances, endogenous factors such as breathing, heartbeat and intrinsic noise in sensory and motor systems cause continual disturbances (Winter, 1995;Forbes et al., 2018).

In the simplest biomechanical description of standing, the body is viewed as an "inverted pendulum" (Gage et al., 2004): the center of mass (CoM), which is the point where the resultant $\vec{G}$ of all the gravity forces acting on the body is applied (Winter, 1995), moves continuously in anterior-posterior and medio-lateral directions with respect to the ankle (Figure S1).

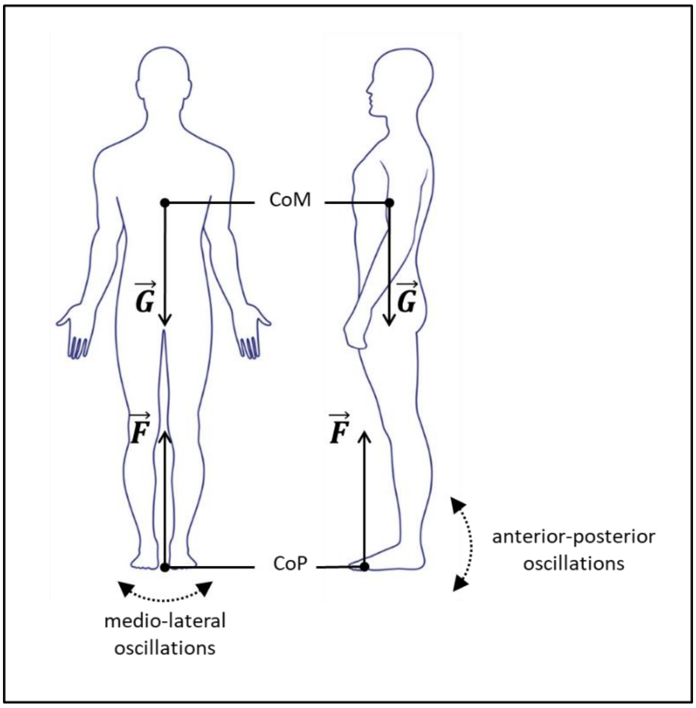

Figure S1 - Biomechanical model of standing posture. Center of mass (CoM); center of pressure (CoP); vertical ground reaction force vector $\vec{F}$; gravitational vector $\vec{G}$.

The oscillations of body posture need to be controlled in order to prevent falling. For this purpose, three major sensory sources are involved: the visual system, the vestibular system, and the somatosensory system; the latter includes a multitude of peripheral sensors (e.g. joint mechanoreceptors, muscle spindles, cutaneous baroreceptors, etc.). The postural controller integrates body information from these multiple sensory sources, and orientates the whole body segment relative to gravitational vector $\vec{G}$ in using peripheral effectors such as muscles that act around the ankle (Forbes et al., 2018).

In practice, a simple and affordable technique to assess postural control is the static posturography, in which individuals are instructed to maintain quiet stance on a fixed support surface (force platform) with their eyes open or closed (Figure S2A). Sway movements of the body are recorded through the trajectory of the center of pressure (CoP) on the force platform, which can be visualized in a horizontal plane with the statokinesigram (Figure S2B).

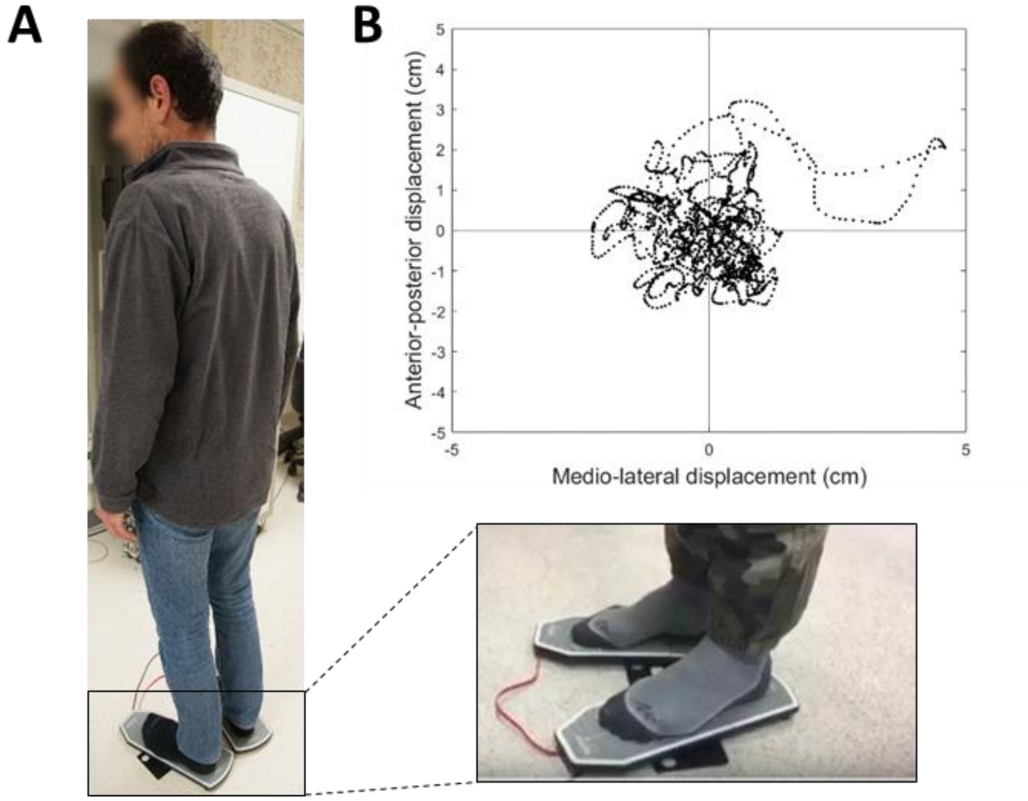

Figure S2 – (A) During posturography individuals are instructed to maintain quiet stance on a fixed support surface (force platform) with their eyes open or closed. (B) Example of statokinesigram that was collected on Eyes closed condition, after data filtering and normalization. The statokinesigram displays the time series of the center of pressure position along medio-lateral (x axis) and anterio-posterior (y axis) axes. It should be noted that origin of axes corresponds to the midpoint between both internal malleolus.

The CoP is the point of application of the vertical ground reaction force vector $\vec{F}$, which is the sum of pressures acting on the part of the body that is in contact with the ground (Winter, 1995). Even though the CoP strictly does not coincide with the projection of the CoM onto the ground (Figure S1), it is usually assumed that the former reflects the latter during quiet standing

(Morasso et al., 1999). Thus, analysis of posturography provides useful insight about the control process of postural balance during quiet standing (Visser et al., 2008;Błaszczyk, 2016).

2) Computation of postural features

This section summarizes the computation of the 16 primary features that were extracted from the postural signal. Data was first low-pass filtered with a 10 Hz cutoff frequency by a fourth-ordered zero-phase-lag Butterworth filter. The values of all 16 postural features were subsequently computed, for each participant and for each condition (Eyes closed and Eyes open) separately (Yamamoto et al., 2015).

Mean-ML and Mean-AP. The features Mean-ML and Mean-AP are the mean positions of the center of pressure (CoP) along medio-lateral (CoP-ML) and anterior-posterior (CoP-AP) axes, respectively. These features are defined as follows:

$$Mean - ML = \frac{1}{P}\sum_{p=1}^{P} \text{CoP-ML}_p \qquad (1)$$

$$Mean - AP = \frac{1}{P}\sum_{p=1}^{P} \text{CoP-AP}_p \qquad (2)$$

where p is the sampling index that ranges from 1 to P.

log-LNG. The feature log-LNG is the log of the total length (in millimeters) of the CoP trajectory on the horizontal plane. This feature is obtained as follows:

$$log - LNG = \log \sum_{p=1}^{P-1} D_p \tag{3}$$

where D_p is the length of the CoP trajectory between two sampling indices (p and $(p+1)$):

$$D_p = \left[\left(\text{CoP-ML}_{p+1} - \text{CoP-ML}_p\right)^2 + \left(\text{CoP-AP}_{\text{p+1}} - \text{CoP-AP}_p\right)^2\right]^{1/2} \tag{4}$$

Zero-cross-V-AP : The instantaneous speed of the CoP is the speed between two sampling times (p and $(p+1)$), which is computed by dividing the distance D_p by the sampling period T = 25ms. The velocity profile depicts the instantaneous speed of the CoP as a function of time.

The feature Zero-cross-V-AP is the total number of events that are defined as the instants of time when the CoP-AP velocity profile crosses zero (Figure S3), where postural data was first low-pass filtered with a 2.5 Hz cutoff frequency.

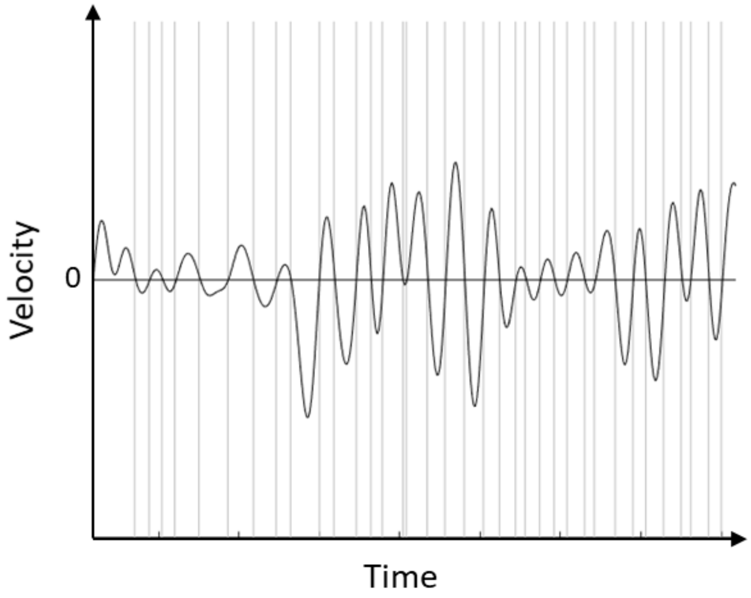

Figure S3 - Velocity profile of the center of pressure along the anterior-posterior axis. Vertical lines represent instants of time when the velocity profile crosses zero. Zero-cross-V-AP is the total number of zero-cross events in the time-span of recording.

log-Alpha-ML, log-Alpha-AP, Beta-ML and Beta-AP. These features are related to the parameters α and β of the probability density function of the Gamma distribution, denoted $p(x)$, which is formulated in general terms as:

$$p(x) = \frac{1}{\Gamma(\alpha)\beta^{\alpha}} x^{\alpha-1} e^{-\frac{x}{\beta}} \qquad (5)$$

where x is the inter-zero-cross interval in the velocity profile, *i.e.* the interval between two zero-cross events (see above definition of the feature Zero-cross-V-AP as example). Specifically, x_{ML} and x_{AP} are the inter-zero-cross intervals in the velocity profiles on ML and AP axes, respectively. The features are defined as follows:

$$log - Alpha - ML = \log \alpha_{ML}$$

$$log - Alpha - AP = \log \alpha_{AP}$$

$$Beta - ML = \beta_{ML}$$

$$Beta - AP = \beta_{AP}$$

MP3 and log-slope-MP. These features are defined using the so-called “sway density”, which is computed by counting the number of consecutive points of the statokinesigram that, for each time instant, fall inside a circle of radius *R* centered at the current CoP point. The sway density curve (SDC) depicts the variations with time of the length of the time intervals (in seconds) during which the CoP trajectory stays within a circle of radius *R* centered at the current CoP location. The SDC often exhibits an oscillatory waveform, which means that the CoP stays around a location for a period of time corresponding to a peak of the oscillatory SDC waveform and then migrates to another location (Figure S4).

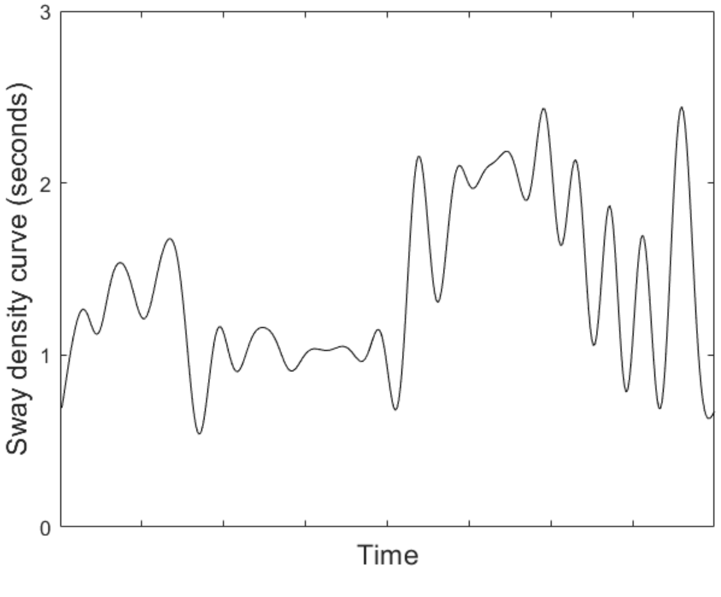

Figure S4 - Sway density curve (SDC) that depicts the variation in time of the length of the time intervals (in seconds) during which the CoP trajectory stays within a circle of radius *R*=3mm centered at the CoP location. The SDC exhibits a regular alternation of peaks and

troughs: the peaks correspond to periods of time in which the CoP stays around a location, and the troughs correspond to periods of time in which the CoP migrates to another location.

After computing the SDC, the peak detection was performed. The MP3 feature is the mean of peak amplitudes (in seconds) of the SDC waveform for R = 3mm:

$$\mathrm{MP3} = \frac{1}{I}\sum_{i=1}^{I} \mathrm{peak}_i \tag{6}$$

where I is the total number of peaks in the time-span of recording, and peak_i denotes the amplitude of the i-th peak of the SDC.

Thus, the larger the value of MP3, the longer the CoP trajectory stays locally. MP indices for other values of R can be obtained similarly (e.g. MP2 for R = 2mm, etc.).

The feature log-Slope-MP is the log of the slope of the line obtained by linear regression of MP *vs*. R from 2 mm to 5 mm.

log-Power and log-Power-ML. The feature log-Power is the log of total power (in mm^2) of the signal that describes the variation in time of the CoP position on the horizontal plane.

The feature log-Power is obtained as the integrated area of the power spectral density function from 0.15 Hz to 5 Hz. Denoting the discrete power spectrum density function by $G(f_m)$, the increment frequency by $\delta f = 0.15$ Hz, the feature log-Power is defined as follows:

$$log - Power = \log \sum_{m=1}^{m=98} G(f_m)\delta f \tag{7}$$

where $f_m = 0.15 + (m-1)\delta f$ and m the increment number that ranges from 1 to 98.

The feature log-Power-ML is the log of the total power of the signal that describes the variation in time of the CoP position along the medio-lateral axis. The feature log-Power-ML is computed similarly to the feature log-Power.

PF95AP. The feature PF95AP is the frequency (in Hz) where 95% of the total power of the signal describing the variation in time of the CoP position along the anterior-posterior axis is found.

The smallest discrete value *v* which satisfies the following equation is calculated:

$$\sum_{m=1}^{m=v} G(f_m)\delta f \geq 0.95 * \text{Power} \tag{8}$$

By using obtained *v*, PF95AP is defined as follows:

$$PF95AP = v\delta f \tag{9}$$

log-MV, log-MV-AP and log-MV-ML. These features are the log of the mean velocity (in mm.sec^{-1}) of the CoP on the horizontal plane, along the anterior-posterior axis and the medio-lateral axis, respectively. They are defined as follows:

$$log - MV = \log \frac{LNG}{P \times T} \tag{10}$$

$$log - MV - AP = \log \frac{1}{P \times T} \sum_{p=1}^{P-1} \left| \text{CoP-AP}_{p+1} - \text{CoP-AP}_p \right| \tag{11}$$

$$log - MV - ML = \log \frac{1}{P \times T} \sum_{p=1}^{P-1} \left| \text{CoP-ML}_{p+1} - \text{CoP-ML}_p \right| \tag{12}$$

where LNG is the total length of CoP trajectory on the horizontal plane (see description of *log-LNG* feature above), p is the sampling index ranging from 1 to *P*, and *T* is the sampling period = 25ms.

Table S1 summarizes the basic statistics of the 16 postural primary features that were extracted from the postural signal.

Table S1 - Means (M) and standard deviations (SD) prior to standardization of the 16 primary postural features in the original sample (N=156), on Eyes closed and Eyes open conditions.

	**Conditions**	
**Postural features (unit)**	*Eyes closed* (M ± SD)	*Eyes open* (M ± SD)
MP3 (sec)	2.17 ± 0.99	3 ± 1.71
Mean-AP (mm)	41.52 ± 18.45	40.51 ± 19.29
Mean-ML (mm)	-1.27 ± 8.53	-1.37 ± 7.54
	§ m: -0.34 ± 7.63; nm: -2.15 ± 9.27	
Zero-cross-V-AP	112.28 ± 15.62	107.89 ± 15.24
Beta-ML	0.14 ± 0.05	0.14 ± 0.05
log-Alpha-ML	0.24 ± 0.18	0.24 ± 0.18
log-Alpha-AP	0.29 ± 0.18	0.26 ± 0.17
Beta-AP	0.17 ± 0.05	0.17 ± 0.05
log-slope-MP	0.20 ± 0.63	0.54 ± 0.69

log-LNG	6.12 ± 0.31	5.91 ± 0.33
log-MV	2.19 ± 0.31	1.97 ± 0.33
log-MV-ML	1.29 ± 0.38	1.24 ± 0.42
log-MV-AP	1.99 ± 0.31	1.70 ± 0.30
log-Power	3.20 ± 1.17	2.95 ± 1.4
log-Power-ML	2.77 ± 0.79	2.76 ± 0.86
PF95AP	1.02 ± 0.28	0.85 ± 0.25

[§] Descriptive statistics of the feature Mean-ML for mindful (m) and non-mindful (nm) individuals, which are intended to contribute to the Discussion.

3) Strategy for data analysis

Regression *vs* classification approaches. The problem that we addressed in the present work was to find the deterministic relation, if any, between the postural signal and the trait mindfulness. To this end, we tested whether features from the postural signal accounted for the FMI score, both being quantitative variables. In addition, the "mindfulness status" (mindful *vs* non-mindful) was computed from the FMI score, thus considering trait mindfulness as a categorical response variable. Hence, depending on the response variable one would consider, *i.e.* the FMI score or the mindfulness status, two distinct problems should be solved: a regression problem and a classification problem, respectively.

Regression approach. The question was: "Can one estimate the FMI score from the postural features, given the available data?". It was assumed that the FMI score y could be appropriately modelled as the sum of the regression function of postural features $f(\mathbf{X})$, which was unknown, and a random zero-mean variable modelling the uncertainty on y. A model was searched in several families of functions $g(\mathbf{X},\boldsymbol{\theta})$ (linear or nonlinear) described below, and the parameters $\boldsymbol{\theta}$ were estimated from the training data. The ultimate goal is to find a model $g(\mathbf{X},\boldsymbol{\theta})$, that is as close as possible to the unknown regression function $f(\mathbf{X})$, given the available data.

Classification approach. The question was: "Can one predict the mindfulness status (mindful or non-mindful) from the postural features, given the available data?". Thus, the classification task was: assign each subject, described by a set of postural features, to one of the two classes defined by the modalities of mindfulness status ("mindful" and "non-mindful"). Solving the classification problem involved two steps: *(i)* estimating the vector of parameters

$\boldsymbol{\theta}$ of the function $g(\mathbf{X},\boldsymbol{\theta})$ that best approximates the posterior probability of the class mindful, *i.e.* the probability that a subject belongs to the class mindful, given his/her set of postural features; *(ii)* assigning the subject to one of the classes, according to the following decision rule: the subject is assigned to the class "mindful" if the posterior probability of that class is larger than a selected threshold t, which is taken equal to 0.5 if false positives and false negatives are equally undesirable.

Linear and nonlinear models. Multiple linear regression and logistic regression were first performed because they are the simplest model for solving regression problems and classification problems, respectively.

Multiple linear regression. Multiple linear regression models how the FMI score y of a subject depends linearly on the vector **X** of the values of the postural features x_j. The model is of the form:

$$g(\mathbf{X},\boldsymbol{\theta}) = \theta_0 + \sum_{j=1}^{F} \theta_j x_j \qquad (13)$$

where $\sum \theta_j x_j$ is a linear combination of postural features x_j, j=1, …, F where F is the number of selected postural features.

In multiple linear regression, the parameters $(\theta_0, \theta_1, \dots, \theta_j)$ are estimated by minimizing a cost function that pictures the "distance" between the model predictions and the measured values for the FMI score. This is done by least squares fitting, which consists in minimizing the cost function (Equation 14) with respect to its parameters:

$$J(\boldsymbol{\theta}) = \sum_{k=1}^{N} \left(y_k - g(\mathbf{X}^k, \boldsymbol{\theta}) \right)^2 \tag{14}$$

where y_k is the trait mindfulness (FMI score) of the *k*th subject of the training set, $\mathbf{X}^k$ is the *k*th vector of postural features, $\boldsymbol{\theta}$ is the vector of model parameters, and *N* is the number of subjects in the training set.

Because multiple linear regression models are linear with respect to their parameters, the least squares cost function is quadratic with respect to them, so that the cost function has a single minimum.

Logistic regression. Logistic regression models the posterior probability that a subject is mindful given the vector **X** of his/her postural features by:

$$p = \frac{1}{1 + e^{-\left(\theta_0 + \sum_{j=1}^{F} \theta_j x_j\right)}} \tag{15}$$

where $\sum \theta_j x_j$ is a linear combination of the subject's postural features x_j.

As mentioned above, the subject is assigned to the class "mindful" if $g(\boldsymbol{X}, \theta) > t$ where t is a threshold chosen by the user, otherwise the subject is assigned to the class "non-mindful". The boundary between the classes in feature space is the locus of points such that $g(\mathbf{X}, \boldsymbol{\theta}) = t$. If $t = 0.5$, $e^{-\left(\theta_0 + \sum_{j=1}^{N} \theta_j x_j\right)} = 1$, so that $\left(\theta_0 + \sum_{j=1}^{N} \theta_j x_j\right) = 0$. The latter relation is the equation of a hyperplane in feature space, so that the classifier obtained by logistic regression is a linear classifier although the model of the posterior probability is nonlinear. The generalization of this result to $t \neq 0.5$ is trivial.

The estimation of the parameters of the model is performed by the maximum likelihood method. Considering a vector of parameters $\boldsymbol{\theta}$, and given the set of observed postural features

for the examples of the training set, the set of estimated posterior probabilities can be computed from Equation 15. The likelihood function is the probability that the postural features and the corresponding classes of the training set can be observed given the parameters. The maximum likelihood method computes the values of the parameters for which the likelihood function is maximum.

Neural Networks. For nonlinear regression, and for the design of nonlinear classifiers, Neural Networks (NN) were used in the present study. As nonlinear models, NNs are particularly attractive to model relations between physiological and psychological phenomena that are very likely to be nonlinear (Cacioppo and Tassinary, 1990). Being nonlinear with respect to both their variables and their parameters, NNs are parsimonious in terms of number of parameters: they require a smaller number of parameters, hence of examples, for a given modelling accuracy, than models that are linear with respect to their parameters, such as polynomials; equivalently, for a given number of parameters and a given number of examples, they have a better modelling accuracy than linear-in-their-parameters models.

We used the simplest form of NN (termed “feedforward neural network” or Multilayer Perceptron), which is a linear combination of nonlinear parameterized functions called “hidden neurons”. It is conveniently represented graphically as shown in Figure S5.

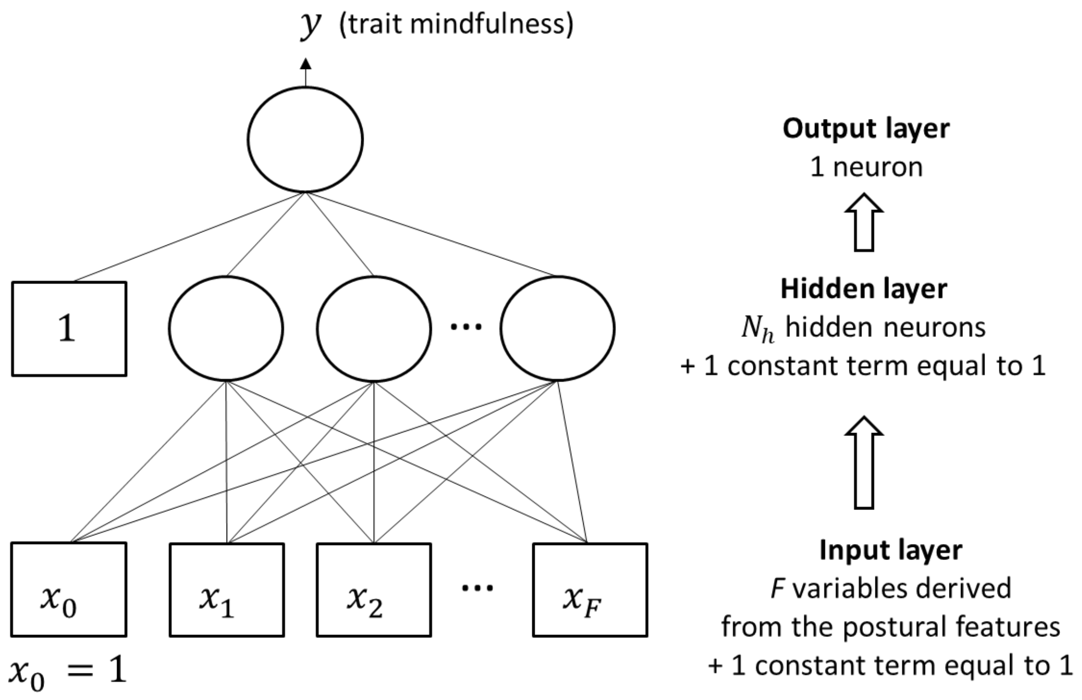

Figure S5 - Graphical representation of a feedforward neural network. In such a network, the information flows from bottom (inputs) to top (output), hence the name "feedforward network". This representation stems from the biological inspiration that prompted the initial interest in formal neurons- (McCulloch and Pitts, 1943;Minsky and Papert, 1969). In the present study, the inputs of the network were the selected variables derived from the postural features of a subject and the output y of the network was his/her Freiburg Mindfulness Inventory score in the regression approach, and his/her mindfulness status (mindful *vs* non-mindful) in the classification approach.

In a feedforward neural network, information flows in the forward direction only, from inputs to output. In the present study, the inputs of the network (shown in squares on

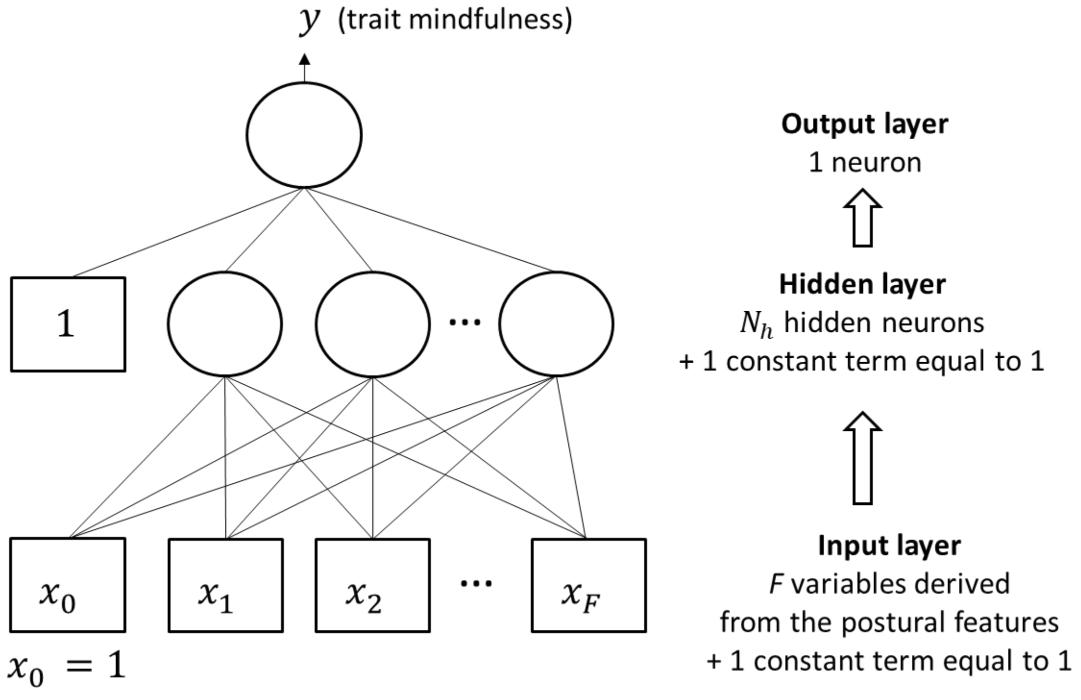

Figure S5) were the F selected variables derived from the postural features, plus a constant term ("bias") equal to 1; the output of the network was the FMI score (in the regression approach) or the mindfulness status (in the classification approach). In the first case, the output of the network was a linear combination of the outputs of the hidden neurons (shown in circles on Figure S5), and of a constant term equal to 1; in the second case, the output of the network was a logistic function of that linear combination.

Each hidden neuron computes a nonlinear function of a linear combination of the network variables. We denote by θ_{ij} the parameter associated to a connection from network input j ($j = 0$ to F) to hidden neuron i ($i = 1$ to N_h where N_h is the number of hidden neurons). Then the output z_i of hidden neuron i is given by

$$z_i = \tanh\left(\sum_{j=0}^{F} \theta_{ij} x_j\right) \tag{16}$$

Denoting by θ_{oi} the parameter associated to the connection between hidden neuron i and the output neuron, the network output is defined, for the regression approach, as follows:

$$y = \sum_{i=0}^{N_h} \theta_{oi} z_i \quad \text{with } z_0 = 1 \tag{17}$$

For the classification approach, the network output is given by

$$y_o = \frac{1}{1 + \exp\left(-\sum_{i=0}^{N_h} \theta_{oi} z_i\right)} \quad \text{with } z_0 = 1 \tag{18}$$

It provides an approximation of the posterior probability of the class of the subject given the postural features.

To find the optimal estimate of their parameters, NN models require the minimization of the cost function $J(\boldsymbol{\theta})$ as described above (Equation 14). Because NNs are nonlinear models with respect to the parameters, the least squares cost function has several minima, one of which must be selected. Therefore, different trainings (with different initial values of the parameters) provide different models corresponding to different minima of the cost function.

4) Feature selection: choice of the threshold in the random probe method

For feature selection, the method used for selecting the set of relevant variables for the estimation of the response variable (FMI score or mindfulness status) was performed in two

steps: *(1)* ranking of the postural features, and pairwise products thereof, in order of decreasing relevance to the response variable using the Orthogonal Forward Regression (OFR) algorithm, and *(2)* rejection of irrelevant features by the random probe method. The latter step is important since keeping irrelevant features is likely to be detrimental to the performance of our models, and rejecting relevant features may be just as bad.

To this end, a set of 100 random features, called “probes”, was appended to the list of candidate postural features; the probes were ranked together with the postural features by the OFR algorithm (see section *Method* for a detailed description of the algorithm (Chen et al., 1989)). Once postural features were ranked, selection was performed: the candidate variables that ranked below a given proportion of the probes (rejection threshold) were discarded. This rejection threshold was computed based on the Cumulative Distribution Function (CDF) of the rank of the probes (as a probe is a random variable, its rank is also a random variable), which allows the model designer to control the risk of selecting a variable although it is irrelevant. The CDF of the rank of the probes was computed by performing 100 iterations of the OFR as follows: at each iteration, one probe was appended to the list of candidate variables; we ran the OFR on a set of (F + 1) candidate variables including the F postural features, their pairwise products, and the probe. That probe was ranked just as the postural features, and its rank was stored. Then, for each rank r (r ranging from 1 to (F + 1)), the cumulative probability of the rank of the probes was estimated as the ratio of the number of probes ranking at, or better than, rank r to the total number of probes.

Figure S6 illustrates the selection of the rejection threshold. Considering the classification approach in Eyes Closed condition, 136 candidate variables were extracted from the postural signal and the CDF of the rank of the probes was computed. Figure S6 shows that the probability that the rank of probes be smaller than or equal to 2 (*i.e.* that one of the best two selected candidate variables be less relevant than one of the probes) is below 0.10. By contrast,

the CDF of the rank of the probes drastically increases from 0.04 to 0.16 for r = 3. Therefore, in this case, the designer should use a threshold of 4% if it is deemed desirable to select the first two features only. By so doing, the designer accepts a 4% risk of selecting a feature although it is weakly relevant or irrelevant. Thus, as in any statistical method, a trade-off must be performed between the risk of designing an oversize model and the risk of designing too small a model.

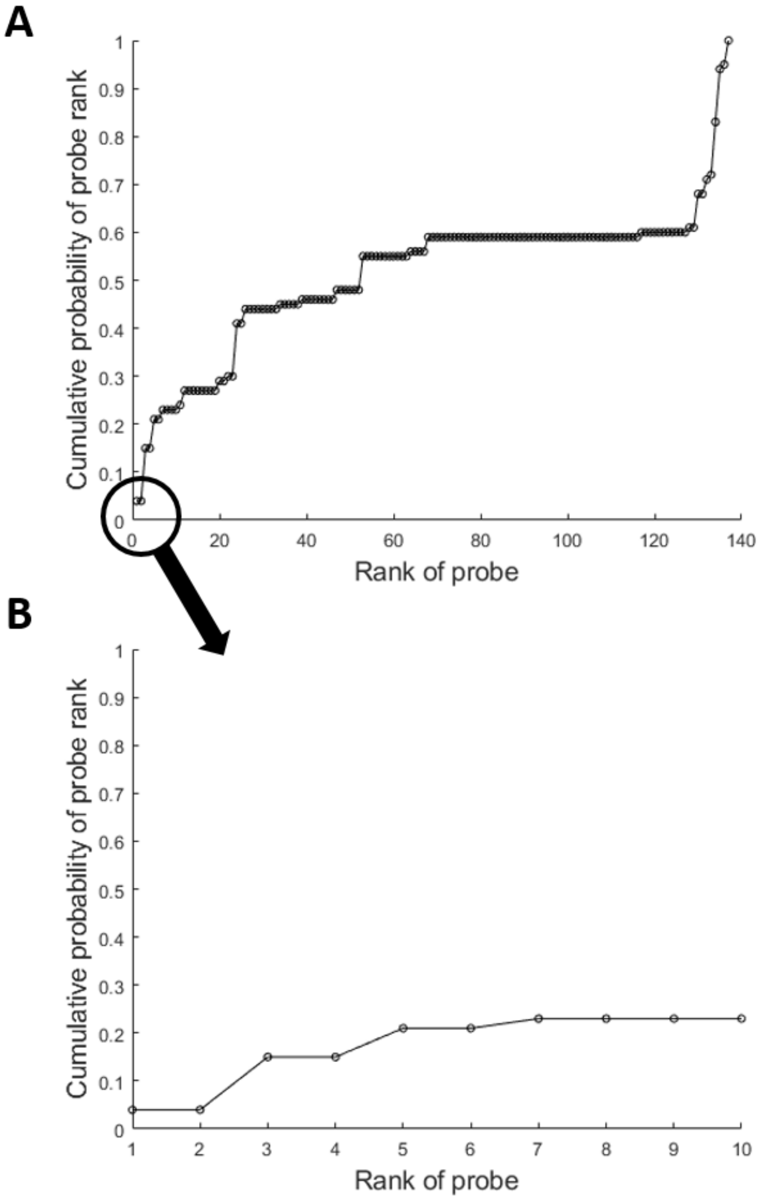

Figure S6 - Cumulative distribution function of the probes **(A)**, with a zoom on the first ten ranks of probes **(B)**.

References

Błaszczyk, J.W. (2016). The use of force-plate posturography in the assessment of postural instability. *Gait & posture* 44**,** 1-6.

Cacioppo, J.T., and Tassinary, L.G. (1990). Inferring psychological significance from physiological signals. *American psychologist* 45**,** 16.

Chen, S., Billings, S.A., and Luo, W. (1989). Orthogonal least squares methods and their application to non-linear system identification. *International Journal of control* 50**,** 1873-1896.

Forbes, P.A., Chen, A., and Blouin, J.S. (2018). "Sensorimotor control of standing balance," in *Handbook of clinical neurology*. Elsevier), 61-83.

Gage, W.H., Winter, D.A., Frank, J.S., and Adkin, A.L. (2004). Kinematic and kinetic validity of the inverted pendulum model in quiet standing. *Gait & posture* 19**,** 124-132.

Mcculloch, W.S., and Pitts, W. (1943). A logical calculus of the ideas immanent in nervous activity. *The bulletin of mathematical biophysics* 5**,** 115-133.

Minsky, M., and Papert, S. (1969). *Perceptrons.* MIT Press.

Morasso, P.G., Spada, G., and Capra, R. (1999). Computing the COM from the COP in postural sway movements. *Human Movement Science* 18**,** 759-767.

Visser, J.E., Carpenter, M.G., Van Der Kooij, H., and Bloem, B.R. (2008). The clinical utility of posturography. *Clinical Neurophysiology* 119**,** 2424-2436.

Winter, D.A. (1995). Human balance and posture control during standing and walking. *Gait and posture* 3**,** 193-214.

Yamamoto, T., Smith, C.E., Suzuki, Y., Kiyono, K., Tanahashi, T., Sakoda, S., Morasso, P., and Nomura, T. (2015). Universal and individual characteristics of postural sway during quiet standing in healthy young adults. *Physiological reports* 3.
